# Tracking Moving Identities: After Attending the Right Location, the Identity Does Not Come for Free

**DOI:** 10.1371/journal.pone.0042929

**Published:** 2012-08-22

**Authors:** Yaïr Pinto, H. Steven Scholte, V. A. F. Lamme

**Affiliations:** Psychonomic Department, University of Amsterdam, Amsterdam, The Netherlands; University of Bologna, Italy

## Abstract

Although tracking identical moving objects has been studied since the 1980's, only recently the study into tracking moving objects with *distinct* identities has started (referred to as Multiple Identity Tracking, MIT). So far, only behavioral studies into MIT have been undertaken. These studies have left a fundamental question regarding MIT unanswered, is MIT a one-stage or a two-stage process? According to the one-stage model, after a location has been attended, the identity is released without effort. However, according to the two-stage model, there are two effortful stages in MIT, attending to a location, and attending to the identity of the object at that location.

In the current study we investigated this question by measuring brain activity in response to tracking familiar and unfamiliar targets. Familiarity is known to automate effortful processes, so if attention to identify the object is needed, this should become easier. However, if no such attention is needed, familiarity can only affect other processes (such as memory for the target set). Our results revealed that on unfamiliar trials neural activity was higher in both attentional networks, and visual identification networks. These results suggest that familiarity in MIT automates attentional identification processes, thus suggesting that attentional identification is needed in MIT. This then would imply that MIT is essentially a two-stage process, since after attending the location, the identity does not seem to come for free.

## Introduction

There are two types of multiple object tracking. You can either track multiple moving objects with identical identities (MOT; for instance, air traffic control), or multiple moving objects, when all objects have distinct identities (MIT; for instance, keeping an eye on your children when they are playing outside).

Most object tracking research up to now has been involved with MOT, the tracking of identical objects [1], [2], [3]. This research has revealed that moving objects can be tracked in parallel [1], [4], with a tracking capacity of, in general, 3–5 objects [5], [6], [7]. In contrast, research into tracking moving objects with a *distinct* identity has been much more limited. MIT research has only started recently [8], [9], [10], although arguably, MIT is ecologically more relevant than MOT (since we are mostly tracking multiple moving objects that are not identical).

This lack of research into MIT so far has obscured many of its main characteristics. For instance, it is unclear how many distinct identities can be tracked. Almost each study finds a different capacity. For instance, Pylyshyn [10] found capacities as low as zero, Horowitz et al. [8] found substantially higher capacities (almost two), and Pinto et al. [11] even higher (approximately three).

A more important mystery surrounding MIT is what neural processes are involved with it. So far, no neural investigation into MIT has been conducted. The search for the neural underpinnings of MIT could also have implications for the functional mechanisms behind MIT. For instance, an important debate is whether MIT consists of two effortful processes or only one. This can be understood as follows. When someone tracks a moving object with a specific identity, she does two things: she attends to the location of the moving object, and she identifies the object at that location. However, the question is: after the location is attended, is it still an effort to identify the object? Or, does the allocation of attention at a specific location, automatically lead to identification of the object as suggested by [8]? Since the difference between these models is crucial for our investigation, we will belabor the point more extensively. The one-stage model assumes one type of attention. So, a subject attends to a location, and then, without further effort or attentional engagement, the object at this location is identified (off course, this only holds when no other interference is occurring such as attentional depletion, or a target that is invisible to the subject). According to the two-stage model two types of attention are involved. First, a location is attended, yet nothing at this location is identified. Second, attention may be employed to identify the object at this location. So, according to the two-stage model, it is possible to attend a location, without identifying the object at that location, which is impossible according to the one stage model. Moreover, and more importantly for the current research, according to the two-stage model, two attentional efforts are needed for MIT (attending to the right location, and then attending to the identity at that location), whereas according to the one-stage model only one attentional effort is required (namely attending to the right location).

In the current study we will compare the tracking of familiar objects to the tracking of unfamiliar objects. So participants will either track moving objects with identities that they know well, or with identities that they are less accustomed to. Note that in the current research familiarity is induced in the same way as in Pinto et al., 2010. That is, pictures (drawn from a larger pool of pictures) are randomly assigned to become familiar or unfamiliar targets. The unfamiliar targets are only designated as a target on 1 trial during the entire experiment, while the familiar targets are designated to be targets on all trials of one run (i.e. on 72 consecutive trials).

An important consequence of tracking a more familiar identity, is that tracking the object becomes easier [11], [12]. This result may be explained in various ways. One possible explanation (as outlined in Pinto, et al., 2010) is that participants do not improve tracking perse, but become better in remembering the target set (which may help retrieve a target after it is lost), or it may reduce the cognitive load since less effort has to be devoted to remembering what the targets are. Note that such explanations are compatible with the one-stage model. Another type of explanation could be along the lines of the two-stage model. Familiarity is known to automate processes [13], [14]. So, perhaps identifying a familiar object is less effortful than identifying an unfamiliar object. Thus, the second stage in the MIT process (attending to the object at a specific location to identify it) becomes less effortful, and therefore MIT on a whole becomes easier. Note that both types of explanations predict the same type of behavioral outcome (MIT becomes easier with familiar objects), but only the latter type of explanations predicts that certain processes should become easier (i.e. less attention would be needed to identify the object). We argue that brain imaging may reveal whether the explanation offered by the two-stage model holds true. If this explanation is correct, then neural activation in areas related to attentional and visual identification should be reduced in the familiar case (because in the familiar case these processes are less effortful). According to the one stage model, in both the familiar and the unfamiliar case attentional and visual identification should be equally effortful so now neural difference in areas related to these processes should be expected.

To sum up, if MIT only requires one effort (namely attending to a location; the one-stage model), then the familiarity of the tracked objects should not cause any neural differences in attentional and visual identification areas. However, if the two stage model is correct we expect to find greater neural activity in attentional [15], [16], [17] and visual identification areas of the brain when one is tracking unfamiliar objects.

To preview our results: we find that the difference between tracking familiar and unfamiliar objects is manifested in several brain areas. The main increase in neural activity, when one is tracking unfamiliar objects, is in two networks: the attention and the visual identification network. Tracking familiar items turns out to also yield increased neural activity in certain areas. Specifically in the resting state network [18], [19], naming and memory areas. We argue that these results support the two-stage model of MIT. That is, to effectively track a moving object with a distinct identity, one needs to first attend the right location, and than employ attention to identify the object at that location.

## Materials and Methods

### Participants

We tested 18 young healthy adults, all with normal, or corrected-to-normal vision. Data from one participant was eliminated from the final fMRI analysis (and excluded from the behavioral analysis), because she produced excessive motion artefacts throughout the experiment. Thus, the final fMRI (and behavioral) sample comprised 17 participants (5 male), ranging in age from 21–32 years old (average 23.7 years).

None of the participants suffered from any condition, currently or in the past that may have negatively impacted brain function. The study was approved by the ethical committee of the University of Amsterdam. Participants were screened before participating, and were only allowed to participate if their participation posed no risks. Participants were paid for participation and provided informed consent before the start of the study.

### Materials

Stimuli were presented on a 24-inch monitor set to a resolution of 1920 by 1200 at a refresh rate of 60 Hz. The experiment was programmed in Matlab 7.5 (The MathWorks) using the Psychophysics Toolbox routines [20], [21]. The output of the monitor was projected on a screen (of 61 cm by 36 cm) by the use of a beamer, with a refresh rate of 240 Hz. Subjects viewed this screen through a mirror, that was 17 cm by 10 cm. The mirror was at a distance of approximately 8 cm from their eyes, and 113 cm from the projection screen. So the effective size of the display was 12° by 8°.

### Design, stimuli and procedure

#### Stimuli

There were three different stimulus-categories, objects, buildings, and faces. Faces were acquired from the face database from the University of Texas at Dallas (https://pal.utdallas.edu/facedb/request/index). We used neutral faces from the age categories 18–69. The stimuli from the categories objects and buildings came from the stimuli database from the Massachusetts Institute for Technology (http://cvcl.mit.edu/MM/stimuli.html). 224 faces (112 male/112 female), 224 buildings, and 224 objects were randomly selected from the stimuli databases to be presented during the tracking task. Furthermore, 13 pictures of buildings, 1 faces (7 female, 7 male) and 14 scrambled faces, were also selected to be used for the localizer task at the end of the experiment.

#### Design

The first part of the experiment consisted of a tracking and a viewing task, intertwined. For both tasks the same stimuli were used. The second and final part consisted of a localizer task.

The first part of the experiment consisted of six runs. Each run tested a different condition. Conditions 1, 2 and 3 denote the ‘familiar’ conditions, 4, 5 and 6 the unfamiliar conditions. A familiar run consisted of 96 trials, an unfamiliar run of 36 trials. Familiar and unfamiliar runs were of unequal length, since familiarity needs many trials to build up, whereas unfamiliarity does not need this. Conditions 1 and 4 employed pictures of buildings; 2 and 5 objects; 3 and 6 faces. Each participant (of a group of six) received a unique order of conditions, according to a latin-square design (with 1 4 2 5 3 6, depicted in table 1, as the first order, and the other five orders as clockwise permutations). In each run one-fourth of the trials were viewing-trials, and three-fourths were tracking trials. The distribution of these viewing-trials was semi-random, i.e. there were always at least two tracking trials separating two viewing trials, but never more than four. Furthermore, the first and the last trial of the run were always viewing trials. Within a familiar run 8 pictures were randomly picked from the pool of 224 pictures. 4 of them were designated as targets, 4 of them as distractors. From trial to trial targets and distractors remained the same. The remaining 216 pictures were used for the unfamiliar run. In this run, target-identities changed every trial, while distractor-identities remained the same throughout the run.

**Table 1:** An example of the experimental design.

Run 1	Run 2	Run 3	Run 4	Run 5	Run 6
Familiar targets; buildings	Unfamiliar targets; buildings	Familiar targets; objects	Unfamiliar targets; objects	Familiar targets; faces	Unfamiliar targets; faces

The experiment consisted of six runs. In each run ¼ of the trials were viewing trials (were participants watched a centrally presented picture), ¾ were tracking trials. Familiar and unfamiliar runs were intertwined. Presented stimuli throughout a run were either buildings, objects or faces. In a familiar trial both targets and distractors remained the same from trial to trial, whereas in an unfamiliar run target-identities changed from trial to trial. Familiar runs consisted of 96 trials, unfamiliar runs of 36 trials. These 6 runs were followed by a localizer task, in which the participant watched faces, buildings or objects.

#### Procedure

Each run started with a 15 second, blank (CIE x-, y- coordinates: . 289, .317, luminance: 63.2 cd/m^2^) screen. During this blank period, subjects were instructed to just view the black fixation spot in the center of the screen (diameter 0.1°).

On a viewing trial a picture of one of the objects presented during the MIT task was flashed three times. Either all flashed pictures were the same, or one of the three flashed pictures was different from the other two. The subject indicated which was the case. If two different pictures were flashed they were either both targets or both distractors from the previous trial, but never mixed (i.e. never one target and one distractor). During an entire viewing trial subjects were instructed to keep their eyes on the center of the screen. The picture was fit to a square of 3.6°×3.6°, and was centrally presented. A viewing trial started with a 0.2 seconds blank screen, followed by three flashes of a picture. Each flash lasted 0.25 seconds. In between flashes, a blank screen was presented for 0.75 seconds. So an entire viewing trial lasted 2.45 seconds. The point of these viewing trials was to measure how object-representations changed as a function of familiarity (however, this is not further reported in this manuscript, but mentioned here to give a comprehensive overview of the task of the participant).

**Figure 1:**
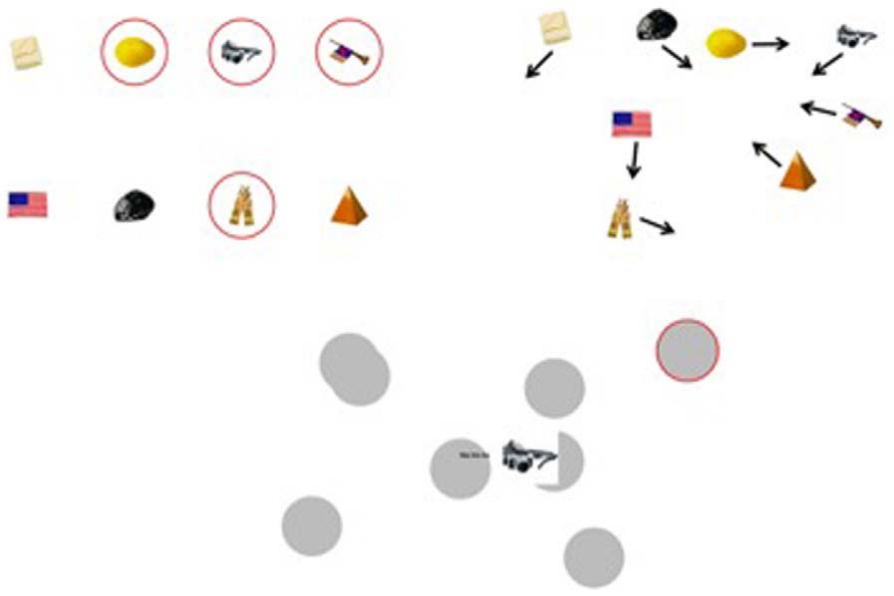
This is a clockwise depiction of a tracking trial. First highlights indicated what the targets are. Then all objects move around for 2–6 seconds. Then all objects are masked, one masked item is highlighted, and the subject indicates if the highlighted item is the same as the centrally depicted probe.

During the entire trial subjects were allowed to move their eyes. A tracking trial started with the presentation of eight pictures (each picture was fitted to a square of 1.24°×1.24°), evenly distributed across an imaginary rectangle (which had a width 12° of and a height of 8° and was centered around fixation). Each target and distractor was assigned (in random order and uncorrelated in order across trials) to one of these eight spots. So when pictures were repeated from trial to trial within a run, the exact location of each picture would randomly vary from trial to trial (although each picture would always start on one of the eight possible spots). It was indicated that an object was a target, by having a red flickering ring around it (diameter 2.07°, width 0.075°, CIE x-, y- coordinates: . 641, .341, luminance: 12.0 cd/m^2^). The flickering lasted one second (with a frequency of 5 Hertz). After this, the pictures remained stationary for a random amount of time between 0 and 3 seconds (with jumps of .5 seconds, so stationary time could be 1.5 seconds, but not 1.3 seconds). So, the encoding part of a tracking trial lasted between 1 and 4 seconds. After the encoding part, the tracking phase started (see Figure 1). The pictures moved around, within the imaginary square, bouncing off the sides of the imaginary square. The targets also bounced off each other, but they passed in front of the distractors. Distractors only bounced off the sides of the imaginary square, passed behind targets, and randomly passed in front or behind of other distractors. The motion phase lasted for a randomly picked time between 2 and 6 seconds. All items moved at a fixed speed throughout the experiment. The speed was determined for each subject individually before the start of the experiment, using a Quest routine [22], [23] (set at 75% correct). Speeds varied between 3.57° and 14.3° per second. During the motion phase subjects were instructed to track the targets. At the end of the motion phase a grey circle (diameter 1.82°, CIE x-, y- coordinates: . 287, .315, luminance: 37.9 cd/m^2^) masked each item. One masked item was highlighted by putting a red ring around it (diameter 2.07°, width 0.075°). Simultaneously a picture was presented in the middle of the screen, and the subject indicated whether the presented picture was the same as the highlighted, masked item. The highlighted, masked item could either be a target or a distractor, the presented picture was always a target. In 1/4 of the cases the highlighted, masked item was a distractor, in 1/3 of the cases both were targets, but not the same targets, in the remaining 5/12 of the cases the presented picture matched the highlighted item. The red ring remained visible until response. If the response was correct the red ring turned green, otherwise it remained red. After response the display (with the color of the ring indicating whether response was correct), remained visible for 0.5 seconds.

In familiar runs, the target-identities and distractor-identities remained the same for the entire run. So, if on the first trial, the subject tracked certain faces, then on subsequent trials, the subject would again track these same faces. In unfamiliar runs target-identities changed from trial to trial (although distractor identities remained the same). So, for instance, if a certain face was a target on one trial, then on a subsequent trial this face could never re-appear again. Nor as target, nor as distractor. Furthermore, target- or distractor-identities from the familiar run would never be used during the unfamiliar run, or vice versa. Per subject it was randomly determined which target- and distractor-identities would appear during the familiar and the unfamiliar runs.

All in all, the completion of the six runs, containing both tracking and viewing trials, took about 1 hour and 50 minutes. In the last 10 minutes of the experiment, subjects performed a localizer task. This localizer task could be later used to determine relevant visual regions (such as FFA: Fusiform Face Area, PPA: Parahippocampal Place Area, and LO: Lateral Occipital area). The localizer task encompassed one run of sixty trials, using the aforementioned stimuli. The trials in the localizer run were the same as the viewing trials except for the following change. In the localizer run the three quickly flashed pictures would always be the same. The task of the subject was to indicate whether the three pictures that were flashed now were the same as the three pictures that were flashed on the previous trial.

Note that, since the experiment took approximately 1 hour and 50 minutes, tiredness and fatigue may have played a role. However, also note that the order of conditions was counterbalanced across subjects, so all conditions were equally affected by this fatigue.

### Data acquisition

The entire experiment was conducted while subjects were placed in the fMRI scanner. We used an event-related design. Since tracking trials waited for the response of the subject, each run of the first part of the experiment did not take a fixed amount of time, but in general, familiar runs lasted about 20 minutes, and unfamiliar runs lasted about 7 minutes (so both familiar and unfamiliar runs had a variable durations, since in both conditions the response of the subject was awaited). The localizer task did take a fixed amount of time (since each trial only had a fixed amount of time within which subjects could respond), and this task took exactly 8 minutes.

In addition to brain activity, we also collected behavioral responses, through the use of button boxes. The main goal of collecting behavioral data was to ensure that subjects paid attention during the viewing trials and the localizer task, and to be able to check whether the ‘familiarity’-manipulation had worked (so the behavioral data should show that subjects were better in tracking familiar than unfamiliar targets).

### Image acquisition

Imaging data were obtained at the University of Amsterdam, Spinoza Center for Functional Magnetic Resonance Imaging using a 3-T Philips scanner using a 8-channel head coil for parallel imaging (SENSE). Foldable foam pads were used to minimize head motion. Echo planar images (EPI) sensitive to the blood oxygen level-dependent (BOLD) effect were obtained with a single-shot gradient echo pulse sequence (TR = 2500 ms; TE = 27.6 ms; FOV = 192×192×125 mm; in-plane voxel resolution = 2×2 mm; SENSE reduction factor = 2; 38 parallel slices; slice thickness = 3 mm). The sequence was planned such that the entire cortex was scanned. A high-resolution T1-weighted 3D sequence was recorded for anatomical reference (160 sagittal slices; TR = 8.1 ms; TE = 3.7 ms; FA = 8°; FOV = 256×256×160 mm; voxel size 1×1×1 mm).

### Image preprocessing

In order to optimize the registration between the different types of image data, non-brain tissue was removed from the structural images using BET [24]. All other image preprocessing was performed using FSL [25]. Structural images were corrected for subject motion artifacts. The functional time-series were first geometrically unwarped and then corrected for subject motion [26] and acquisition delay between slices.

The functional images were spatially smoothed with a Gaussian filter (5-mm full width at half maximum (FWHM) and resliced into 2-mm isotropic voxels. The data were high pass filtered. Furthermore, low frequencies were cut at 0.01 Hz. Registration of the functional images to standard space was performed in a two-step procedure ([26] and [27]). Bold-MRI was registered to the high-resolution structural scan with 7 degrees of freedom and the resulting transformation matrix was then applied to the functional image that was aligned to the reference image. Finally, the high-resolution structural scan was registered to a standard brain with 12 degrees of freedom followed by through non-lineair warping.

### Statistical analyses

The expected signal time courses of every subject from each run were modeled using a box-car function that was convoluted with a double gamma hemodynamic response function [28].

To test for regional differences in activation between the familiar and unfamiliar condition, we set up an analysis with two predictors, “familiar” and “unfamiliar” (based on whether the participant was tracking familiar or unfamiliar targets), and estimated the effect of condition (i.e., familiar>unfamiliar and unfamiliar>familiar) with voxelwise *t*-tests. For single subject results, a fixed effects model was used, for the group results, we used a mixed effects model.

Time-series statistical analysis was carried out using FILM with local autocorrelation correction [29]. Data were pooled over runs within subjects by forcing the random effects variance to zero. Higher level analysis over subjects was carried out using FLAME (FMRIB's local analysis of mixed effects) stages 1+2 ([30] and [31]). We corrected for multiple comparisons by using cluster thresholding with a z-value of 2.3 (i.e. p<0.01, subsequently a p<0.05 was used: so p<0.01 was set at the voxel-level for cluster-size estimation, and then a cluster-level corrected treshold of 0.05 was applied. Cluster based tresholding was based on Gaussian Random Field Theory, controlling at the cluster level for family wise errors. Note that this is somewhat liberal, thereby increasing the chance of type I errors, i.e. false positives).

## Results

**Figure 2:**
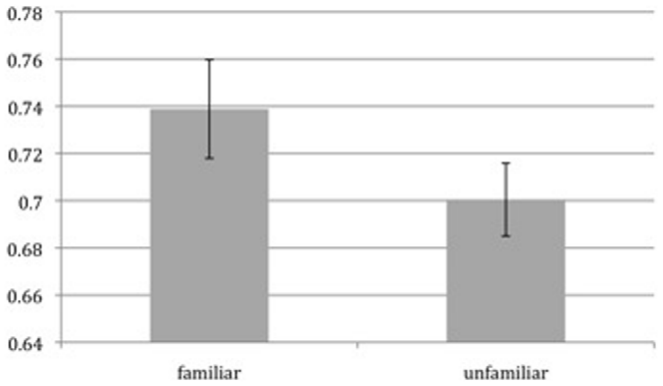
Behavioral data showing that subjects performed significantly better, with regards to identity accuracy, on familiar trials. On the y-axis percentage correct is indicated.

**Figure 3:**
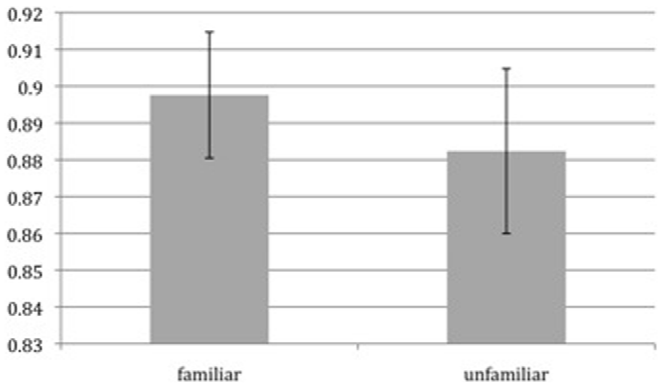
The behavioral data shows no significant improvement for location accuracy.

Our research question is what neural processes underlie MIT. We tackle this question by contrasting tracking familiar with unfamiliar objects. First we checked whether our familiarity-manipulation was effective. We compared tracking accuracy in the familiar condition to accuracy in the unfamiliar condition, see Figures 2 and 3. We looked at two types of accuracy: identity and location accuracy. Identity accuracy denotes whether participants knew about the identity of a target, whereas location accuracy denotes whether they knew the location of a target (identity performance was measured by only analysing those trials when a target location was highlighted, when identity knowledge was necessary for responding correctly; location performance was measured by analysing those trials where a distractor location was highlighted, in which case knowledge about the location of the targets would suffice to answer correctly). This revealed a significantly better performance in the familiar condition (t(16) = 2.6, p = 0.02), when it comes to identity accuracy. When it came to location accuracy, we observed no significant difference between familiar and unfamiliar trials (p>0.4, t<1) The behavioral data demonstrate that our familiarity manipulation was effective (at least when it came to tracking identities), and we again show, in replication of Oksama and Hyönä (2008) and Pinto et al. (2010), that subjects are better at tracking familiar than unfamiliar items.

However, note that the difference in accuracy between familiar and unfamiliar is rather small. This could be due to the fact that the distractors played a smaller role than usual in this set-up. Pinto et al. (2010) found that when distractors were as visible as targets, there was a familiary effect (difference between familiar and unfamiliar accuracy) of almost 20%. However, when distractors were removed, this effect was reduced to approximately 5%. So, perhaps, because in the current set-up targets were always visible, and moved in front of distractors, the familiarity effect was reduced.

We proceeded to investigate what change in brain activity underlies this change in performance. We contrasted brain activity in the familiar condition to brain activity in the unfamiliar condition. Before we discuss the results of these comparisons we should make a note of caution. As mentioned, encoding time varied per trial, this may have introduced variance. Furthermore, the difference in duration between the familiar and unfamiliar runs (although the order of these runs was counterbalanced) is another possible source of unwanted variance. Both these manipulations may limit any conclusions we draw based on comparisons between the familiar and unfamiliar condition.

**Figure 4:**
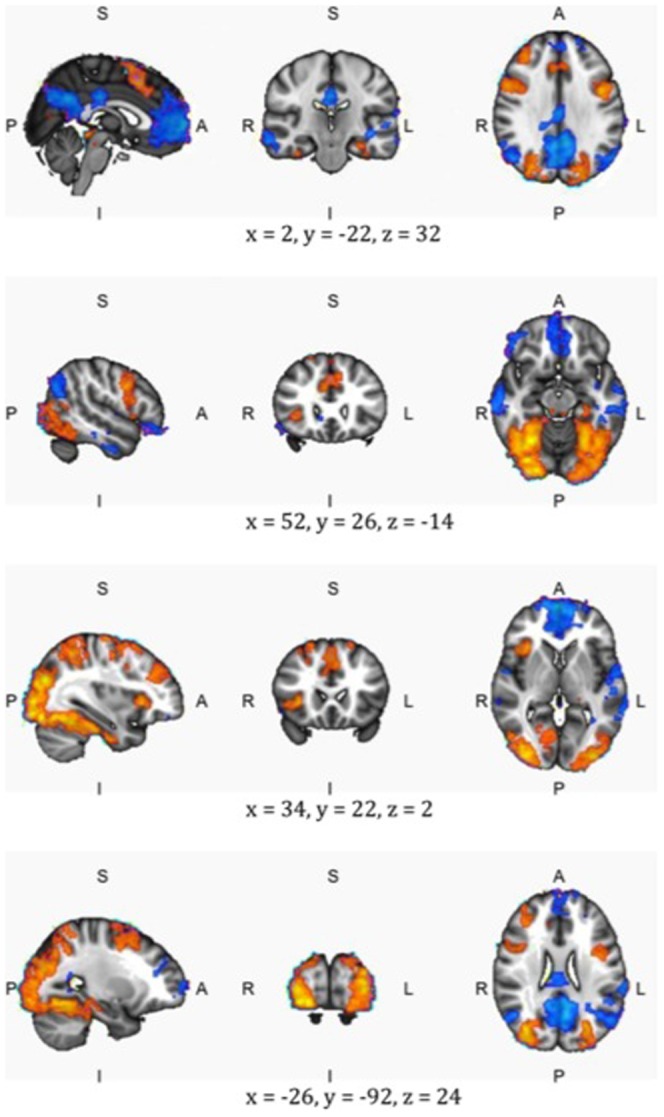
A side, sliced and top-view of four points in the brain: Blue shows the areas that are more active during the familiar condition and red those areas that are more active during the unfamiliar condition. Center of gravity coordinates (MNI reference system) are shown below each slice. The left picture shows the side view of the brain (s indicates the top of the brain, I the bottom, p the back, and a the front). The middle picture shows a sliced view of the brain (r denotes the rights side of the brain, l the left side). The right picture shows a top view of the brain. For more brain slices: see Appendix S1.

We found increased activity in the default state network during familiar trials. Higher brain activity in the unfamiliar runs was found in the occipital and ventral visual areas, and in the attentional network. See Figure 4, Appendix S1 and S2 and tables 2 and 3 for an overview of the differences in brain activity between familiar and unfamiliar trials.

**Table 2:** An overview of the regions that are more active during unfamiliar trials.

Unfamiliar>familiar							
Name	Hemi	Voxels	Cluster sign.	z-max	X	Y	Z
Occipital pole	right	8925	<10?−34	5.6	32.7	−66.1	3.5
Occipital pole	left	7257	<10?−29	5.33	−31.6	−71.9	2.24
Middle frontal gyrus	right	4298	<10?−20	4.23	15.2	8.29	49.9
Precentral gyrus	left	572	<.001	4.36	−44.9	4.03	30.5
Middle frontal gyrus	right	449	<.005	3.41	36.5	39.6	31.3
Insular Cortex	right	272	<.05	4.02	39.1	21.2	−0.0482

Coordinates are in mm. One voxel is 2×2×3 mm. Names are based on the Harvard-Oxford cortical structural atlas. LOC stands for lateral occipital cortex.

**Table 3:** A similar overview as in Table 2, but now of the regions that are more active during familiar trials.

Familiar>Unfamiliar							
Name	Hemi	Voxels	Cluster sign.	z-max	X	Y	Z
Frontal pole	bilateral	4995	<10?−22	4.62	−0.249	55.3	4.3
Superior temporal gyrus, posterior division	left	3862	<10?−18	4.47	−55.1	−37.9	7.91
Precuneous cortex	bilateral	2672	<10?−14	4.96	−0.947	−58.6	25.9
Middle temporal gyrus, posterior division	right	858	<10?−5	3.71	62.4	−20.4	−12.7
LOC, superior division	bilateral	668	<0.0001	3.77	51.2	−64.8	31.9
Cingulate gyrus, posterior division	right	411	<0.005	3.97	3.96	−24.8	30
Frontal pole	left	344	<0.05	3.58	50.4	37.2	−14.2

### Increased activity in the unfamiliar condition

See Table 2. More detailed information about the increased activity in the unfamiliar condition can be seen in the slices displayed in appendix S1, and the list of local maxima in appendix S2. We found increased activity in the unfamiliar condition in the right middle frontal gyrus, the precentral gyrus and the right insular cortex. The observed increase in activity in these areas in the unfamiliar condition seems to fit well with the goal-directed attentional network [15], [16], [17]. We also found increased activity in the occipital pole. The increased activity in this area is part of a visual network, involved with perceiving and recognizing objects [32], [33], [34], [35], [36].

Thus, it may be that tracking unfamiliar trials places higher demands on attentional identification of targets. This then leads to increased neural activity in two neural networks: those associated with attentional processes and those underlying visual identification processes.

### Increased activity in the familiar condition

See Table 3, and again, for more details, see appendix S1 and S2.

In the familiar condition we found increased activity in the superior temporal gyrus (posterior division), the Middle temporal gyrus (posterior division), the frontal pole, the Cingulate gyrus (posterior division), the precuneous cortex and the LOC, superior division.

We hypothesize that the increased activity in the the middle temporal gyrus, and the superior temporal gyrus, may be due to increased memory (MTG, [37], [38]) and increased naming (STG, [39], [40]) activity. We argue that it may be that this reflects an increased effort of participants to memorize the targets in the familiar run (also by naming them), since they knew that in the familiar run targets would repeat.

Furthermore, the frontal pole, the Cingulate gyrus (posterior division), the precuneous cortex and the LOC, superior division are thought to be part of the ‘resting-state’ network [18], [19]. Thus, it could be that, since both encoding and tracking at some point during the run became easier in the familiar case, participants started mindwandering, which caused an increase in activity in the resting-state network. Note that the task, even in the familiar case, never became very easy. However, the task became *easier* than in the unfamiliar condition, and, we argue, therefore mindwandering increased. Note furthermore that mindwandering is not the only possible interpretation of the increased activity in these areas. Other functions, such as memory retrieval and spatial working memory have also been associated with this network, and perhaps the increased neural activity reflects more emphasis on these functional mechanisms [41], [42], [43], [44], [45], [46].

## Discussion

In the current study we investigated which neural processes underly MIT. We researched this question by contrasting the tracking of familiar objects with the tracking of unfamiliar objects, since we expect automation of attentional identification in the familiar condition. However, if no attentional identification occurs during MIT (as claimed by the one-stage model), then we should find no traces of such automation. Importantly, we found neural evidence supporting automation of attentional identification of targets. We found increased activity in the attentional network, and in the visual identification areas, in the unfamiliar condition. In the familiar condition, we found increased memory, naming and resting-state activity.

Importantly, a goal of this study was to unveil whether MIT is a one-stage process or a two-stage process. The implications of answering this question may go beyond MIT. It may tell us something essential about attention. In this regard, the data are suggestive. Familiarity seems to automate processes related to attentional identification. Or to rephrase: after attending a location, it seems that attending to the identity of that object is still required (i.e. the identity does not come for free after attending the right location). Thus, MIT seems to be a two-stage process.

### Attention in MIT

A first key point to note is that our results indicate that the ‘standard’ goal-directed attentional areas play a role in MIT. It may seem trivial that this is the case, however, at least with regards to MOT, this is not the case. The influential FINST-theory of Pylyshyn [2], [47], states that it is not the case that each moving object is attended, but that each object receives an index (or as Pylyshyn calls it, a ‘Finger of INSTantiation’). This index could be regarded as some kind of inferior type of attention, essentially different from the standard goal-directed type of attention (such as used in visual search tasks).

Importantly then, the current study shows that, at least in MIT, the standard goal-directed type of attention does seem to play an important role in multiple object tracking. Note that this may be the case because MIT seems to require attending to the identity of an object. It may still be that just attending to a location does not activate the standard goal-directed attentional network.

### Two stage process

The current results suggest that tracking unfamiliar objects places higher demands on attentional *and* visual identification processes. If in both the familiar and the unfamiliar case visual identification would come for free once a location is attended, then this increased toll on attentional identification would not be expected. Thus, the present data support to a two-stage model of MIT. This is in line with the results of Pylyshyn (2004), Horowitz et al. (2007), and Pinto et al. (2010). All of these researches found that location capacity is higher than identity capacity. This implies that a location can be known, without knowing what resides at that location, suggesting that attending the location is not enough to know which object is there.

Importantly, the two stage-model seems somewhat at odds with the influential Feature Integration Theory [48]. FIT suggests that attending a location immediately releases (and binds) all the features at that location. According to such a notion, it could be expected that when one is attending a moving object, the identity of that moving object should come for free. Note that in MIT, it may be that participants focus on multiple locations in parallel. If that is the case, it may be that FIT holds when there is only one focus of attention (i.e. in that case attention to a location does imply that all features at that locations are bound into one identity), but that FIT does not hold when there are multiple foci of attention (and therefore, in an MIT task a second stage, involving attention to the identity at a location, is required).

Furthermore, the two-stage model may also place some limits on the reversed hierarchy model [49]. The final percept of the subject does not seem to be an entirely processed package containing both location and identity. It seems more like a partially processed product, which could contain location, but only partial identity information.

### Familiarity and MIT

Although in the current study familiarity has been used as a tool it is an interesting phenomenon by itself. How is it that becoming more familiar with an object makes it easier to track? Both Oksama & Hyönä [12] and Pinto et al. [11] have researched this question. Pinto et al. found that the familiarity benefit is not due to better memory for the target-set or prevention of target-distractor confusion, which (among other findings) led them to conclude that familiarity facilitates the tracking process. The question then becomes, how is tracking improved by familiarity?

The current study suggests an answer. It seems that familiarity improves both the allocation of attention, and the identification of objects at the attended location. This may be understood as follows. Perhaps, when engaged in a demanding task, the visual system does not fully process the viewed objects. Perhaps, after some features are processed, a quick decision is made about the identity of the object. And the more familiar with the object, the quicker this decision is reached. So, with an unfamiliar object, the visual system processes more details before deciding what identity is at a location. After familiarizing, it may be that this decision is reached quicker (so, with an unfamiliar object, after detecting that the element has several vertical appendices, is more or less rectangular in shape, and is gray, the visual system may decide that this must be an elephant; however when this same elephant becomes familiar, it may be that just noticing that the object is gray may be enough to conclude that the object is an elephant).

A prediction of this hypothesis would be that increased familiarity would in fact *decrease* the likelihood of detecting an unexpected change to the item. The more familiar the item, the less irrelevant features are encoded.

### Conclusion

MIT seems to consist of two effortful stages, first attending to the right location, and then attending to the identity at this location. This is the so-called two-stage model. It seems that just attending a location is not enough to know what is at that location. This conclusion may challenge popular views on attention, that claim that attending a location immediately releases the identity at that location.

Furthermore, we speculate that these results point to a fundamental feature of how identities are observed by the visual system. It may be that the visual system selects locations, and identifies some key features at this location. These features are then linked to internal ‘flags’ informing the system about what is where. When the visual system becomes more familiar with an object, the flags become simpler, making it easier to locate (and thus track) that object.

## Supporting Information

Appendix S1 **An overview of relevant brain slices.** Center of gravity coordinates (MNI reference system) are shown below each slice. The left picture shows the side view of the brain (s indicates the top of the brain, I the bottom, p the back, and a the front). The middle picture shows a sliced view of the brain (r denotes the rights side of the brain, l the left side). The right picture shows a top view of the brain.
(DOCX)

Appendix S2 **Local maxima per cluster.**
(DOCX)
