# Supplementary material for: Tracking Moving Identities: After Attending the Right Location, the Identity Does Not Come for Free
Source: PLoS One. 2012 Aug 22;7(8):e42929. doi: 10.1371/journal.pone.0042929 (PMC3425545; doi:10.1371/journal.pone.0042929)
Supplement: Appendix S1 — An overview of relevant brain slices. Center of gravity coordinates (MNI reference system) are shown below each slice. The left picture shows the side view of the brain (s indicates the top of the brain, I the bottom, p the back, and a the front). The middle picture shows a sliced view of the brain (r denotes the rights side of the brain, l the left side). The right picture shows a top view of the brain. (DOCX) [file pone.0042929.s001.docx]

Appendix 1: an overview of relevant brain slices

Center of gravity coordinates (MNI reference system) are shown below each slice. The left picture shows the side view of the brain (s indicates the top of the brain, I the bottom, p the back, and a the front). The middle picture shows a sliced view of the brain (r denotes the rights side of the brain, l the left side). The right picture shows a top view of the brain.
