# Supplementary material for: Tracking Moving Identities: After Attending the Right Location, the Identity Does Not Come for Free
Source: PLoS One. 2012 Aug 22;7(8):e42929. doi: 10.1371/journal.pone.0042929 (PMC3425545; doi:10.1371/journal.pone.0042929)
Supplement: Appendix S2 — Local maxima per cluster. (DOCX) [file pone.0042929.s002.docx]

Appendix 2: Local maxima per cluster:

**Fam > Unfam**

| **Cluster** | **Local maximum Z** | **x** | **y** | **z** | **name** | **hemi** |
| --- | --- | --- | --- | --- | --- | --- |
| frontal pole | 4.62 | 0 | 66 | 0 | frontal pole | bilateral |
| frontal pole | 4.62 | -4 | 58 | -4 | frontal pole | bilateral |
| frontal pole | 4.24 | 0 | 60 | -4 | frontal pole | bilateral |
| frontal pole | 4.16 | 8 | 58 | -2 | frontal pole | bilateral |
| frontal pole | 4.09 | -4 | 62 | 2 | frontal pole | bilateral |
| frontal pole | 4.03 | 14 | 68 | 4 | frontal pole | Bilateral |
| Superior temporal gyrus, posterior division | 4.47 | -70 | -30 | 22 | Supra marginal gyrus | left |
| Superior temporal gyrus, posterior division | 4.33 | -68 | -32 | 16 | Superior temporal gyrus, posterior division | left |
| Superior temporal gyrus, posterior division | 4.06 | -62 | -34 | 18 | Planum temporale | left |
| Superior temporal gyrus, posterior division | 3.98 | -66 | -36 | 22 | Supra marginal gyrus | left |
| Superior temporal gyrus, posterior division | 3.8 | -58 | -68 | 20 | LOC, superior division | left |
| Superior temporal gyrus, posterior division | 3.78 | -50 | -70 | 28 | LOC, superior division | left |
| Precuneous cortex | 4.96 | 4 | -72 | 26 | Cingulate gyrus, posterior division | bilateral |
| Precuneous cortex | 4.75 | -8 | -54 | 26 | Cingulate gyrus, posterior division | bilateral |
| Precuneous cortex | 4.63 | -8 | -64 | 28 | Precuneous cortex | bilateral |
| Precuneous cortex | 4.55 | -8 | -52 | 32 | Cingulate gyrus, posterior division | bilateral |
| Precuneous cortex | 4.5 | -4 | -74 | 26 | cuneal cortex | bilateral |
| Precuneous cortex | 4.25 | 6 | -52 | 30 | Cingulate gyrus, posterior division | bilateral |
| middle temporal gyrus, posterior division | 3.71 | 68 | -26 | -14 | middle temporal gyrus, posterior division | right |
| middle temporal gyrus, posterior division | 3.66 | 62 | -34 | -10 | middle temporal gyrus, posterior division | right |
| middle temporal gyrus, posterior division | 3.6 | 60 | -14 | -22 | middle temporal gyrus, posterior division | right |
| middle temporal gyrus, posterior division | 3.44 | 60 | -16 | -12 | middle temporal gyrus, posterior division | right |
| middle temporal gyrus, posterior division | 3.32 | 64 | -28 | -8 | middle temporal gyrus, posterior division | right |
| middle temporal gyrus, posterior division | 3.31 | 64 | -12 | -10 | middle temporal gyrus, posterior division | right |
| LOC, superior division | 3.77 | 48 | -70 | 34 | LOC, superior division | right |
| LOC, superior division | 3.6 | 48 | -60 | 22 | LOC, superior division | right |
| LOC, superior division | 3.6 | 48 | -56 | 26 | Angular gyrus | right |
| LOC, superior division | 3.58 | 50 | -66 | 34 | LOC, superior division | right |
| LOC, superior division | 3.41 | 52 | -68 | 28 | LOC, superior division | right |
| LOC, superior division | 3.39 | 56 | -66 | 26 | LOC, superior division | right |
| Cingulate gyrus, posterior division | 3.97 | 2 | -22 | 32 | Cingulate gyrus, posterior division | bilateral |
| Cingulate gyrus, posterior division | 3.58 | 18 | -36 | 30 | Callosal body | bilateral |
| Cingulate gyrus, posterior division | 3.51 | 0 | -22 | 38 | Cingulate gyrus, posterior division | bilateral |
| Cingulate gyrus, posterior division | 3.4 | 6 | -22 | 26 | Cingulate gyrus, posterior division | bilateral |
| Cingulate gyrus, posterior division | 3.11 | 0 | -28 | 24 | Cingulate gyrus, posterior division | bilateral |
| Cingulate gyrus, posterior division | 2.99 | 6 | -30 | 34 | Cingulate gyrus, posterior division | bilateral |
| Frontal pole | 3.58 | 52 | 26 | -14 | Frontal orbital cortex | right |
| Frontal pole | 3.53 | 50 | 42 | -18 | frontal pole | right |
| Frontal pole | 3.33 | 52 | 46 | -14 | frontal pole | right |
| Frontal pole | 3.25 | 46 | 50 | -14 | frontal pole | right |
| Frontal pole | 3.12 | 52 | 32 | -18 | Frontal orbital cortex | right |
| Frontal pole | 3.07 | 58 | 32 | -6 | Inferior frontal Gyrus | right |

**Unfam>Fam**

| **Cluster** | **Local maximum Z** | **x** | **y** | **z** | **name** | **hemi** |
| --- | --- | --- | --- | --- | --- | --- |
| Occipital pole | 5.6 | 32 | -94 | -12 | Occipital pole | right |
| Occipital pole | 5.48 | 32 | -94 | -16 | Occipital pole | right |
| Occipital pole | 5.37 | 28 | -56 | -16 | Temporal occipital fusiform cortex | right |
| Occipital pole | 5.29 | 28 | -62 | -12 | Occipital fusiform gyrus | right |
| Occipital pole | 5.28 | 28 | -66 | -12 | Occipital fusiform gyrus | right |
| Occipital pole | 5.27 | 34 | -80 | 24 | LOC, superior division | right |
| Occipital pole | 5.33 | -26 | -92 | 24 | Occipital pole | left |
| Occipital pole | 5.32 | -34 | -88 | 16 | LOC, superior division | left |
| Occipital pole | 5.05 | -28 | -60 | -12 | Temporal occipital fusiform cortex | left |
| Occipital pole | 4.88 | -38 | -68 | -18 | Occipital fusiform gyrus | left |
| Occipital pole | 4.8 | -34 | -92 | 2 | Occipital pole | left |
| Occipital pole | 4.75 | -34 | -92 | 8 | Occipital pole | left |
| Middle frontal gyrus | 4.23 | 38 | 0 | 58 | Middle frontal gyrus | right |
| Middle frontal gyrus | 4.17 | 24 | 4 | 68 | Superior frontal gyrus | right |
| Middle frontal gyrus | 4.06 | 2 | 18 | 46 | Paracingulate gyrus | right |
| Middle frontal gyrus | 4.02 | 56 | 8 | 34 | Precentral gyrus | right |
| Middle frontal gyrus | 3.96 | 26 | 8 | 54 | Superior frontal gyrus | right |
| Middle frontal gyrus | 3.94 | -34 | -2 | 60 | Middle frontal gyrus | left |
| Precentral gyrus | 4.36 | -42 | 4 | 30 | Precentral gyrus | left |
| Precentral gyrus | 3.36 | -40 | 12 | 26 | Inferior frontal gyrus | left |
| Precentral gyrus | 3.36 | -36 | 12 | 28 | Middle frontal gyrus | left |
| Precentral gyrus | 3.25 | -54 | 8 | 34 | Precentral gyrus | left |
| Precentral gyrus | 3.06 | -40 | 2 | 20 | Precentral gyrus | left |
| Precentral gyrus | 3.05 | -44 | 0 | 20 | Precentral gyrus | left |
| Middle frontal gyrus | 3.41 | 36 | 34 | 32 | Middle frontal gyrus | right |
| Middle frontal gyrus | 3.38 | 30 | 42 | 26 | Frontal pole | right |
| Middle frontal gyrus | 3.38 | 36 | 40 | 34 | Frontal pole | right |
| Middle frontal gyrus | 3.18 | 34 | 32 | 28 | Middle frontal gyrus | right |
| Middle frontal gyrus | 3.18 | 38 | 52 | 26 | Frontal pole | right |
| Middle frontal gyrus | 3.18 | 36 | 38 | 22 | Frontal pole | right |
| Insular cortex | 4.02 | 34 | 22 | 2 | Insular cortex | right |
| Insular cortex | 3.93 | 38 | 18 | 0 | Insular cortex | right |
| Insular cortex | 3.9 | 42 | 18 | -2 | Insular cortex | right |
| Insular cortex | 3.83 | 38 | 26 | -2 | frontal orbital cortex | right |
| Insular cortex | 2.98 | 52 | 14 | 0 | Inferior frontal gyrus | right |
